# Differential effects of L-tryptophan and L-leucine administration on brain resting state functional networks and plasma hormone levels

**DOI:** 10.1038/srep35727

**Published:** 2016-10-20

**Authors:** Davide Zanchi, Anne Christin Meyer-Gerspach, Claudia Suenderhauf, Katharina Janach, Carel W. le Roux, Sven Haller, Jürgen Drewe, Christoph Beglinger, Bettina K. Wölnerhanssen, Stefan Borgwardt

**Affiliations:** 1 Department of Psychiatry, University Hospital of Basel, CH-4012 Basel, Switzerland; 2 Department of Biomedicine, University Hospital, CH-4031 Basel Switzerland; 3 Diabetes Complications Research Centre, Conway Institute University College Dublin, Dublin, Ireland; 4 Faculty of Medicine of the University of Geneva, Switzerland; 5 Affidea CDRC – Centre Diagnostique Radiologique de Carouge, Switzerland; 6 Department of Surgical Sciences, Radiology Uppsala University, Uppsala, Sweden; 7 Department of Neuroradiology, University Hospital Freiburg, Germany; 8 Faculty of Medicine of the University of Geneva, Switzerland; 9 Department of Research, St. Claraspital, Switzerland

## Abstract

Depending on their protein content, single meals can rapidly influence the uptake of amino acids into the brain and thereby modify brain functions. The current study investigates the effects of two different amino acids on the human gut-brain system, using a multimodal approach, integrating physiological and neuroimaging data. In a randomized, placebo-controlled trial, L-tryptophan, L-leucine, glucose and water were administered directly into the gut of 20 healthy subjects. Functional MRI (fMRI) in a resting state paradigm (RS), combined with the assessment of insulin and glucose blood concentration, was performed before and after treatment. Independent component analysis with dual regression technique was applied to RS-fMRI data. Results were corrected for multiple comparisons. In comparison to glucose and water, L-tryptophan consistently modifies the connectivity of the cingulate cortex in the default mode network, of the insula in the saliency network and of the sensory cortex in the somatosensory network. L-leucine has lesser effects on these functional networks. L-tryptophan and L-leucine also modified plasma insulin concentration. Finally, significant correlations were found between brain modifications after L-tryptophan administration and insulin plasma levels. This study shows that acute L-tryptophan and L-leucine intake directly influence the brain networks underpinning the food-reward system and appetite regulation.

Luminal enteral communication is a key factor in the regulation of appetite, food intake and metabolism. Protein digestion to dipeptides or tripeptides and free amino acids modulate digestive functions, glycemia and appetite^1^,^2^,^3^,^4^,^5^,^6^. Protein is currently believed to exert the greatest appetite-suppressing effect of the three macronutrients (carbohydrates, fats and proteins) in animals and humans^7^. High-protein diets have been extensively studied for their ability to reduce total energy intake and body weight^7^. Mechanisms that have been suggested include stimulation of insulin release^3^, postprandial thermogenesis^1^, intestinal gluconeogenesis^7^, and direct effects of amino acids in regions of the brain^6^. In addition, it has been hypothesized that protein-induced satiation could be due to alterations in the release of gastrointestinal satiation peptides, such as cholecystokinin (CCK), glucagon-like peptide-1 (GLP-1) and peptide tyrosine tyrosine (PYY). Already in 1956, it was suggested that an elevated concentration of plasma amino acids serve as a satiation signal for food intake and thereby results in depressed food intake^8^. To date the effect of specific amino acids on satiation and appetite is only rarely studied.

Amino acids, including L-leucine^9^,^10^, L-glutamine^11^,^12^, and L-phenylalanine^13^ modulate appetite and/or glycemia in lean, obese, or type 2 diabetic subjects. The aromatic amino acid, L-tryptophan, is of particular interest, as previous studies have reported effects on digestive functions^14^ and food intake^15^.

It is unknown how amino acids affect specific brain regions. After eating, the brain senses a biochemical change and then signals satiation, but the precise sequence of events has not been determined. Even for established physiological systems such as glucose-insulin regulation, the timing of the interaction between hormonal processes and neural events has mostly been inferred from blood sampling studies. Recently, neuroimaging studies have provided *in vivo* information about the neuro-anatomical correlates of the regulation of energy intake. Temporal orchestration of such systems is, however, crucial to the integration of the neural and hormonal signals that control eating behaviour. In a landmark paper demonstrating eating-related neural activity in the brain, the response was shown to interact with an internal signal, plasma insulin^16^. As it was shown that most amino acids induce an increase in insulin (possibly due to an increase in GLP-1), amino acids could be one of the signals from the gut that interact with the brain through satiation peptides.

The present study was designed to further investigate the luminal influences of nutrients, which orchestrate gut-brain interactions. The objective was to compare the effects of intragastric L-tryptophan (L-Trp) and L-leucine (L-Leu) on brain networks and the release of insulin and glucose. We used plasma levels to represent physiological differences; this is a reliable measure of appetite and satiety^17^,^18^. In particular the association between brain activity in areas involved in appetite, appetite and satiety rating scores and levels of insulin and glucose was already demonstrated in previous studies^16^,^19^.

On the basis of previous studies highlighting the differences between L-Trp and L-Leu on digestive functions^20^, with L-leucine stimulating appetite and L-tryptophan stimulating satiety^21^, we hypothesized that L-Trp would induce a different activation pattern in the brain, leading to modifications in brain networks related to appetite and metabolism regulation. The selection of the doses of L-tryptophan was based on the daily intake recommended by World Health Organization (WHO)^22^. The doses are comparable to the amount of L-tryptophan in soybeans in a normal portion of an Asian dish. For L-leucine an isocaloric approach to L-tryptophan was chosen. Glucose was chosen as a positive control and water as a negative control as we have previously shown effects on brain activity with both of them^23^. We have never intended to do an isocaloric comparison between glucose and amino acids.

## Results

### Physiological and psychological results

#### Differences in insulin levels

**Figure 1:**
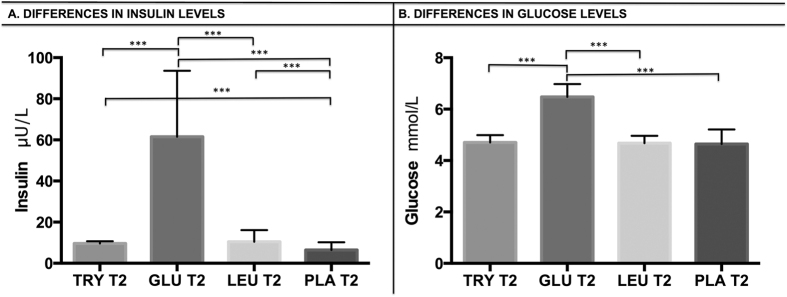
Physiological results. After treatment administration, plasma hormones levels were compared though a paired t test. (**A**) Higher insulin concentrations were found after glucose ingestion than with L-tryptophan (p < 0.001), L-leucine (p < 0.001) or placebo (p < 0.001). Moreover, after L-tryptophan and L-leucine administration, significantly higher insulin levels were found than with placebo (p < 0.001). No statistical differences were found between insulin levels after L-tryptophan and L-leucine intake. (**B**) Higher glucose levels were found after glucose than with L-tryptophan (p < 0.001), L-leucine (p < 0.001) or placebo (p < 0.001). No significant differences in glucose levels were found between other treatments.

(Figure 1A) No significant differences were found between the treatments in insulin levels at baseline (Time1, before treatment). At Time2, the ANOVA was significant (p < 0.001) showing significant differences in insulin plasma levels after the treatments administration. In particular, significantly higher insulin concentrations were found after glucose administration than with L-tryptophan (p < 0.001), L-leucine (p < 0.001) or with placebo (p < 0.001). Significantly higher insulin levels were found after L-tryptophan and L-leucine administration than with placebo (p < 0.001). No statistical differences were found between insulin levels after L-tryptophan or L-leucine administration. Cohen’s effect size (d): d = 2.37, var(d) = 0.17, p < 0.001.

#### Differences in glucose levels

(Figure 1B) At baseline (Time1, before treatment), no significant differences in glucose levels were found between the treatments At Time2, the ANOVA was significant (p < 0.001) showing significant differences in glucose plasma levels after the treatments administration. In particular, significantly higher glucose levels were found after glucose than after L-tryptophan (p < 0.001), after glucose than after L-leucine (p < 0.001) and after glucose than after placebo (p < 0.001). No significant differences in glucose levels were found between the other treatments. Cohen’s effect size (d): d = 3.66, var(d) = 0.27, p < 0.001.

### Functional connectivity results

From framewise displacement (FD) analyses, no significant effect of motion was found between the treatments.

At baseline (Time0), after permutation based non-parametric tests, no significant activation was found in the three networks for any group comparison, revealing that, before medication, there were no differences between the subjects in functional connectivity.

At Time2, after permutation based non-parametric tests, ANOVA showed significant differences between the treatments within each of the three pre-selected networks (p < 0.001). Cohen’s effect size (d) for the default mode network was: d = 1.97, var(d) = 0.15, p < 0.001. Cohen’s effect size (d) for the sensorimotor network was: d = 2.72, var(d) = 0.23, p < 0.001. Cohen’s effect size (d) for the saliency network was: d = 1.07, var(d) = 0.17, p < 0.001.

**Figure 2:**
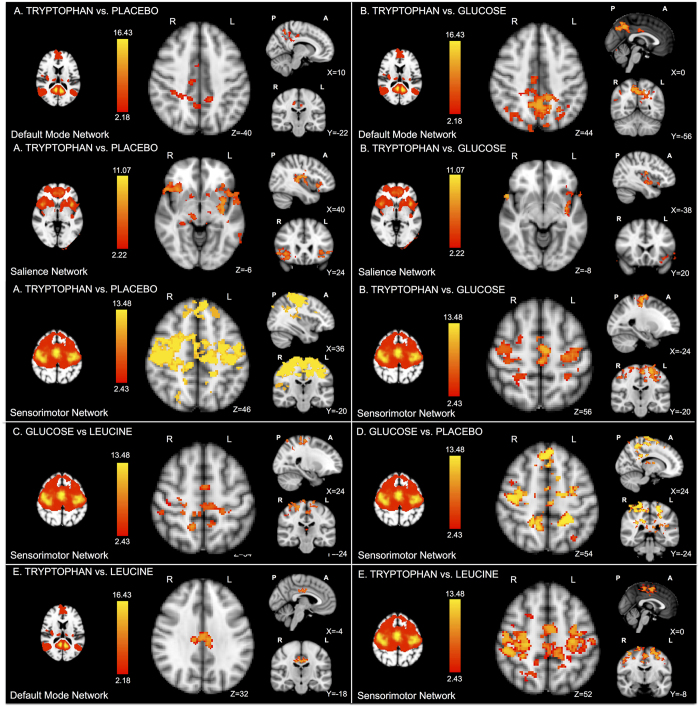
Functional connectivity results. After independent component analyses (ICA), the dual regression technique was performed to investigate differences between the different treatments in functional connectivity in the DMN, SN and SMN. (**A**) The comparison “L-tryptophan vs. placebo” revealed increased connectivity in the cingulate cortex and in the precuneous within the default mode network (DMN), in the somatosensory cortex within the sensorimotor network (SMN) and in the bilateral anterior insula within the salience network. (**B**) The comparison “L-tryptophan vs. glucose” showed altered connectivity in areas overlapping those of the previous contrast. In particular, the cingulate cortex and the precuneous show higher connectivity within the DMN, the left anterior insula within the SN and the somatosensory cortex within the SMN. (**C**) The comparison “glucose vs. L-leucine” revealed significantly higher connectivity in the bilateral sensorimotor cortex within the sensorimotor network. (**D**) Within the same network (SMN), the comparison “glucose vs. placebo” reveals an increase in connectivity in additional areas, including the precuneous. (**E**) The comparison “L-tryptophan vs. L-leucine” revealed significantly increased connectivity in the cingulate cortex within the DMN and in the somatosensory cortex within the SMN. No significant activations where found for the remaining comparisons. These results reveal that amino acids administration has an extensive influence on the brain, by modifying the connectivity of specific functional networks related to appetite perception and emotional regulation.

In particular, the comparison “L-tryptophan vs. placebo” revealed increased connectivity in the cingulate cortex and in the precuneous within the default mode network (DMN), in the somatosensory cortex within the sensorimotor network (SMN) and in the bilateral anterior insula within the salience network (SN) (Fig. 2A).

For the comparison “L-tryptophan vs. glucose”, group analyses showed reduced connectivity in areas overlapping those in the “L-tryptophan vs. placebo” contrast. In particular, the cingulate cortex and the precuneous show higher connectivity within the DMN, the somatosensory cortex within the SMN and the left anterior insula within the SN (Fig. 2B).

With respect to the sensorimotor network (SMN), the comparison “glucose vs. L-leucine” revealed significantly higher bilateral connectivity in the sensorimotor cortex (Fig. 2C), while the comparison “glucose vs. placebo” shows broader connectivity in areas including the precuneous (Fig. 2D).

The comparison “L-tryptophan vs. L-leucine” revealed significantly increased connectivity in the cingulate cortex within the DMN and in the somatosensory cortex within the SMN (Fig. 2E).

No significant activations where found for the remaining comparisons: “glucose vs. L-tryptophan”, “placebo vs. L-tryptophan”, “placebo vs. L-leucine”, “placebo vs. glucose”, “L-leucine vs. L-tryptophan”, “L-leucine vs. glucose” and “L-leucine vs. placebo”.

Finally, the interaction effect between time and treatments revealed no significant results.

These results reveal that amino acid administration has extensive effects on the brain, by modifying the connectivity of specific functional networks.

### Correlations between imaging and physiological results

After L-tryptophan administration, a positive correlation (p < 0.05) was found between insulin plasma levels and bilateral activity in the insula within the saliency network (Fig. 3.1). Furthermore, after glucose administration, positive correlations were present between plasma insulin levels and activity in the sensorimotor area (SMA) within the sensorimotor network (SMN) (Fig. 3.2). These results established a link between the role of satiety hormones and connectivity changes in the brain after amino acid administration.

### VBM Analysis of T1 Images

VBM analysis of the grey matter (GM) revealed no statistical differences between the visits at baseline.

## Discussion

The current study provides novel insights into the effects of luminal amino acids on gut-brain interactions. The present findings suggest that intragastric L-tryptophan (L-Trp) and L-leucine (L-Leu) lead to differential modifications in insulin and glucose plasma concentrations and in brain networks connectivity, that are related to metabolic regulation and appetite sensations.

Our results from laboratory analyses indicate that different amino acids affect specific satiation hormones. In particular, L-tryptophan and L-leucine lead to a significant increase in insulin plasma levels when compared to water, but at the doses given they do not affect glucose levels. The differential effects of amino acids on insulin but not on glucose have been shown before in several studies^11^. On the other hand, and in line with previous studies^24^, glucose is associated with both insulin and glucose plasma levels. As L-Trp and L-Leu have been proposed to be involved in food-intake and regulation of energy homeostasis^25^,^26^,^27^, our findings suggest that L-tryptophan and L-leucine are key amino acids that affect satiety and appetite perception. To further explore these dynamic interactions, we investigated the effects of these amino acids on brain functional networks that are related to the food-reward mechanism.

Our additional findings are related to large-scale alterations in brain networks involved in metabolic regulation after amino acids ingestion. Our results reveal that - compared to glucose and placebo - L-tryptophan gives higher connectivity within the default mode network in the cingulate cortex and in the precuneous and within the salience network in the bilateral insula. In general, the finding that activity in brain areas regulating appetite can be influenced by different nutrients is consistent with previous reports^19^,^28^ on modifications after glucose and fructose ingestion. Our study extends this research, by focusing for the first time on amino acids ingestion and suggesting that L-tryptophan may be a key amino acid that increases brain connectivity in areas controlling the metabolic state of the individual.

Moreover, the direct link between satiation hormones and brain areas involved in metabolism regulation is confirmed by the positive correlation between brain activity in the insular cortex and insulin plasma levels after L-tryptophan administration. These results resemble previous findings in studies with glucose intake and confirms that there is a direct link between these regions and food-reward mechanisms^16^.

Furthermore, in comparison to placebo and glucose, L-tryptophan administration also increases activity within the sensorimotor network in somatosensory areas. This is the first study to report large-scale modifications in brain activity after L-tryptophan manipulation within the sensorimotor network. The reorganization in the sensorimotor network is also evident in the analysis of the effects of glucose administration compared to placebo that shows greater activation in somatosensory areas. As demonstrated in a previous study^16^, the somatosensory area (SMA) integrates sensory and visceral signals associated with protein intake and is therefore one of the main brain areas involved in appetite regulation and food-reward mechanism. In our study, its function is confirmed by the positive correlation found between changes in activity in SMA and in insulin.

Apart from brain modifications related to the food-reward system, we infer that L-tryptophan has an influence on cognitive functions and mood regulation. In fact, as demonstrated by different studies manipulating serotonin levels, changes in the activity in prefrontal regions can affect cognitive control and emotion processing^29^,^30^,^31^,^32^. In particular, as suggested by Kramer^33^, L-tryptophan depletion is linked to reduced activity in the insula, that in turn regulates decision making in potential aggressive situations. On the other side, modulation of L-tryptophan leads to changes in the DMN that may reduce depressive mood^34^. Following this interpretation changes in the DMN and SN after L-tryptophan intake can be linked to changes in cognitive functions and emotion processing.

Our last result concerns the role of L-leucine on brain networks at rest. When L-tryptophan was compared to L-leucine, no differences were found in the activity in the insular cortex within the SN or within the DMN in the precuneous, while significant differences were present in the ACC within the DMN and within the SMN in the SMA. Moreover, comparison of glucose vs. L-leucine didn’t find any differences in the DMN or SN.

Even if no previous studies have been conducted on the effects of L-leucine at brain level, these results suggest that this amino acid has an effect on brain network connectivity that is not equivalent to that of water, which suggests that L-leucine may influence areas responsible for cognitive and metabolic processing. Further investigations are needed to further clarify the effect of this amino acid on brain networks at rest.

It is important to note that this study has some limitations. As in previous neuroimaging studies of the brain-gut axis in healthy subjects, our sample size was modest because the design of the study makes recruitment of subjects relatively difficult. On the other hand Cohen’s effect size analyses reveal that the treatments show already high magnitude. In addition, the present study focused on only two amino acids. Further investigations should be conducted on more amino acids. Moreover, plasma levels were measured for glucose and insulin, while other hormones might have better detected physiological differences between the different amino acids. Our results might potentially be influenced by mood variations not investigated by the oral examination of the health status of the participants. However, it is important to highlight that the aim of the present study is to investigate changes in brain networks involved in satiation and appetite regulation and changes in hormones levels also related to satiation and appetite (as glucose and insulin). Moreover, it is unlikely that there are systematic changes in mood associated with specific amino acids (the treatments were randomized), hence it is unlikely that potential mood variations systematically biased the current results. Furthermore, the fMRI examination in our study is not conditioned by a paradigm, so the results reflect pure resting state functional connectivity and they may not be comparable to other studies that use a tasks-related approach. Concerning the fMRI analyses, it is important to notice that smoothing may introduce spurious local functional connectivity and affect the subsequent conduction of ICA. Therefore, we performed the analyses without applying smoothing and we compared the three functional networks with and without smoothing. As expected smoothing improves the quality of the results, making the components less noisy. Therefore we decided to work on smoothed data. Lastly, T1 images were acquired at Time0 but not Time2, but the preprocessing of the Time2 images used the T1 image at Time0, so there were different space templates in segment. Since the subjects stayed in the MRI during the treatments administration, the position of the subjects remained the same during all the sequences, therefore the registration can be considered quite effective. Moreover, we repeated the analyses registering the EPI images directly to standard space. Slightly worse registration was seen in the brain networks for the registration directly to MNI, as expected (in fact this is the reason why the high-resolution 3DT1 was used). Despite this small reduction in the quality of the spatial normalization, we observed no relevant differences in the brain networks identified by ICA between the two different registrations. This shows that the registration first to T1 and then to MNI was the most effective one, and that the effect of the spatial registration on the results was marginal.

Finally, it is important to underline possible confounding results from structural changes in grey matter that can influence the response to the treatment administration. To avoid this, VBM analyses were conducted before treatment administration and no differences were observed between the visits, thus excluding this potential confounder.

## Conclusion

The current work provides new insights into acute neural modifications after ingestion of amino acids. By linking satiety hormones and fMRI measurements, this study shows that acute L-tryptophan and L-leucine intake directly affect specific brain networks that underpin the food-reward system and appetite regulation.

## Materials and Methods

### Participants

The protocol was approved by the Ethics Committee of Basel, Switzerland (EKBB: 08/11) and conducted in accordance with the principles of the Declaration of Helsinki. All experimental procedures were carried out in accordance with the approved guidelines. All participants gave written informed consent prior to inclusion. Twenty-three (23) subjects were recruited through local and Internet advertising. Each participant underwent a medical interview, laboratory screening and gave written informed consent. Exclusion criteria were: lactose intolerance, smoking, substance abuse, regular intake of medications, medical or psychiatric illness, and any contraindication to MRI (e.g. claustrophobia, non-removable metal devices) or abnormalities detected upon laboratory screening. Of the 23 subjects originally recruited, three had to be excluded, as they did not meet the eligibility criteria. The final sample included 20 healthy volunteers (28.1 ± 6.2 years, 11 females). Estimation of statistical power in functional MRI requires knowledge of the expected percent signal change between two conditions, as well as estimates of the variability in percent signal change. We calculated the sample size for a strict statistical adjusted threshold of p < 0.05, 20 subjects were required to achieve 50% power at the single voxel level for brain activations in the a priori defined networks of interest.

### Experimental Protocol

This was a randomized, placebo-controlled, double blind, crossover study and was carried out at the Phase I Research Unit of the University Hospital of Basel. L-tryptophan, L-leucine, glucose and placebo were administered to each subject on four different days, following the procedure described below. The treatment order was randomized and at least 7 days passed between the visits. Therefore no interaction was present between the four administrations.

The subjects started each physiological and imaging examination between 9 and 10am in the morning, after an overnight fast of at least 10 hours. The subjects consumed no breakfast before the visits.

**Figure 4:**
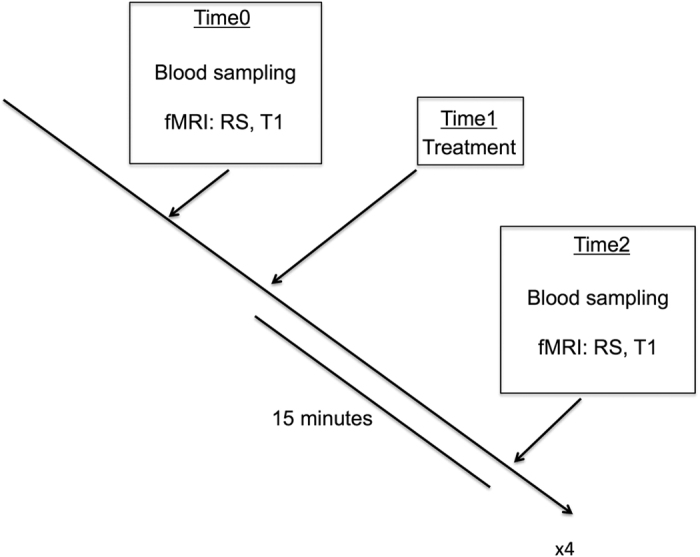
Study design. After an overnight stay of at least 10 hours, the study was carried out in three phases: Baseline (Time0), treatment administration (Time1) treatment assessment (Time2). The baseline examination (Time0) was conducted to control for possible differences in hormone levels and brain functions and structures before treatment administration. Two blood samples were taken through a peripheral venous cannula and stored for laboratory analyses (for blood collection method refer to 38. To assess for possible functional and structural differences at the brain level, the subjects underwent an fMRI examination, including a functional resting state (RS) sequence and a T1 sequence. After the fMRI examination, the treatment was administered (Time1). An 8F polyvinyl nasogastric tube was inserted into the stomach through an anaesthetized nostril. Subjects received 300 ml tap water with 1.56 g (7.5 mmol) L-tryptophan, 75 g glucose, 1.56 g (11.89 mmol) L-leucine and 300 ml pure tap water (placebo) via the nasogastric tube over 2 minutes while sitting in the MR room. 15 minutes after administration, the tube was removed. To evaluate treatment effects, the subjects underwent a second physiological and brain imaging examination (Time2): blood sampling assessed through a peripheral venous cannula and fMRI examination (RS sequences) were repeated. No T1 sequence was used in this phase, since structural changes were not expected in the short period of time after treatment administration.

The study was carried out in three phases: Baseline (Time0), treatment administration (Time1) treatment assessment (Time2) (Fig. 4).

Before each visit health status assessment was performed orally by a physician. At the beginning of the experiment an 8F polyvinyl nasogastric tube was inserted into the stomach through an anesthetized nostril and its intragastric position was confirmed by rapid injection of 10 ml of air and auscultation of the upper abdomen. Then two blood samples were taken through a peripheral venous cannula and stored for laboratory analyses. Baseline examination (Time0) was then conducted, in order to control for possible differences in hormone levels and brain functions and structures before treatment administration. To assess for possible functional and structural differences at the brain level, subjects underwent an fMRI examination, including a functional resting state (RS) sequence and a T1 sequence. Before scanning a pillow in dotation of the MRI PRISMA was set in the head-coil behind the head of the subjects to prevent subjects head movements.

After the fMRI examination, the treatment was administered (Time1). The solutions were freshly prepared and were at room temperature when administered. L-tryptophan and L-leucine were purchased from Sigma Aldrich Chemical Company, Germany (>97% pure) and glucose monohydrate was purchased from Haenseler AG (Herisau, Switzerland). Different persons prepared and administered the treatment, in order to maintain the double blindness of the study. Subjects received 300 ml tap water with 1.56 g (7.5 mmol) L-tryptophan, 1.56 g (11.89 mmol) L-leucine, 75 g glucose, and 300 ml pure tap water (placebo) via a nasogastric tube, over 2 minutes while sitting in the MR room.

15 minutes after administration, the tube was removed. To evaluate treatment effects, the subjects underwent a second physiological and brain imaging examination (Time2): blood sampling assessed through a peripheral venous cannula and fMRI examination (RS sequence) was repeated. No T1 sequence was used in this phase, since structural changes were not expected, due to the short period of time after treatment administration.

### Laboratory analyses

*Plasma glucose* concentration was measured by a glucose oxidase method (Rothen Medizinische Laboratorien AG, Basel, Switzerland). The intra- and inter-assay coefficients of variation are below 2.9% and below 3.9%, respectively.

Plasma Insulin was measured with a commercially available electrochemiluminescence immunoassay (Cobas/Roche Diagnostics GmbH, Mannheim, Germany). The intra- and inter-assay coefficients of variation for this assay are below 2.0% and below 2.8%, respectively.

### fMRI acquisition

Images were obtained using a 3T scanner (Prisma; Siemens, Erlangen, Germany) with a standard 32-channel head-coil. fMRI imaging of the whole brain was acquired by echo planar imaging, using the following parameters: whole brain coverage, TR = 1.8 s, TE = 28 ms, 35 slices, slice thickness 3.5 mm, 168 repetitions. The 3D T_1_-weighted structural scan had the following parameters: 256 × 256 matrix size, 176 sections, 1 × 1 × 1 mm^3^, TE = 3.37 ms, TR = 2000 ms).

### Statistical Analysis

The statistical analyses were conducted using GraphPad Prism (Version 6, GraphPad Software, San Diego, USA), FSL (Version 5.0.9, FMRIB, Oxford, UK) and R (Version 0.99.896, The R-Project for Statistical Computing).

### Analysis of physiological data

To compare hormones levels between the different treatments at Time1 and Time2 separately, a repeated measure analysis of variance (ANOVA) was performed with Tukey correction for post-hoc pair-wise comparisons. Cohen’s effect size was also calculated using compute.es package in R (https://cran.r-project.org/) to assess the strength of the difference in hormones plasma levels between the treatments.

### Functional connectivity analysis

#### Pre-processing of functional data

Processing and analysis of imaging data of Time1 and Time2 were performed using FSL. Preprocessing included brain extraction using FSL’s BET (Brain Extraction Tool), motion correction using FSL’s MCFLIRT (intra-modal motion correction tool)^35^, spatial smoothing of 5 mm using FSL’s SUSAN (noise reduction uses nonlinear filtering)^36^. High-pass temporal filtering of 100 seconds was used according to the standard MELODIC ICA procedure in FSL (http://fsl.fmrib.ox.ac.uk/fsl/fslwiki/MELODIC). Functional images were first co-registered to structural images (acquired during Time1) using linear transformation and later normalized to MNI space using linear transformation. In addition, for each subject, we computed a maximum of the framewise displacement^37^,^38^ from the realignment parameters and subjected this to group (treatments) comparison (ANOVA).

### RSNs and Subcortical structures identification

To define brain networks at rest, independent component analysis (ICA) was carried out on the resting state data of Time1 using FSL’s multi-session multivariate exploratory linear optimized decomposition into independent components (MELODIC Multi-session temporal concatenation)^39^. First automatic estimation of components was used to explore resting state networks, but due to high parcellation of the signal, the number of components was set to 25 as suggested by previous studies and as common practice in ICA for fMRI data^40^. Out of these 25 components, we decided to select and focus our analyses on 3 resting-state networks (RSNs) identified as consistent by previous studies^41^,^42^ and involved in appetite regulation and control of metabolism^43^: Default Mode Network (DMN), Sensorimotor Network (SMN) and Saliency Network (SN).

### RSNs group comparison and correlations with clinical scores

A dual regression approach with nonparametric permutation (5000) tests (randomize, FSL) was carried out on the resting state data at Time1 and separately at Time2 to detect statistically significant differences between treatments (placebo vs. L-tryptophan, L-tryptophan vs. glucose, glucose vs. L-leucine, etc.) within the boundaries of the three RSNs identified at Time1. A repeated measure ANOVA was performed.

Results were corrected for multiple comparisons using threshold free cluster enhancement (TFCE) and p values < 0.05 were considered as significant. TFCE is similar to cluster-based thresholding, but generally more robust and avoids the need for the arbitrary initial cluster-forming threshold. It is recommended when randomize is performed^44^. Moreover, we performed an ANOVA comparing the visits in respect to time to test whether any interaction effect between times and treatments is present. Finally, Cohen’s effect size was also calculated for each comparison within each network using compute.es package in R (https://cran.r-project.org/).

### Correlations between imaging and physiological results

Additionally, we tested for possible correlations between regions that were significantly active in the previous contrasts and hormones levels.

On the basis of the results of the dual regression, we defined regional masks and extracted region-averaged time courses of each subject for the three resting state networks. These values were then correlated with the insulin and glucose levels of each subject, using Pearson correlations.

### VBM Analysis of T1 Images

To assess differences in grey matter density between groups, a voxel-based morphometric (VBM) analysis was performed in FSL (FSL Version 5.0.9; http://fsl.fmrib.ox.ac.uk), using standard processing steps^45^,^46^. Firstly, BET extraction and tissue-type segmentation were performed using the corresponding FSL tools (Brain Extraction Tool and FAST4). Secondly, non-linear transformation into Montreal Neurological Institute (MNI) reference space was applied and a study-specific grey matter (GM) template was created. The native GM images were then non-linearly registered to this template. Finally, the images were smoothed with an isotropic Gaussian kernel of 2 mm sigma. A voxel-wise GLM was implemented using permutation-based nonparametric testing (Randomise, part of FSL). Results were corrected for multiple comparisons using TFCE^44^ and p values < 0.05 were considered as significant.

## Additional Information

**How to cite this article**: Zanchi, D. *et al*. Differential effects of L-tryptophan and L-leucine administration on brain resting state functional networks and plasma hormone levels. *Sci. Rep.* **6**, 35727; doi: 10.1038/srep35727 (2016).

## Figures and Tables

**Figure 3:**
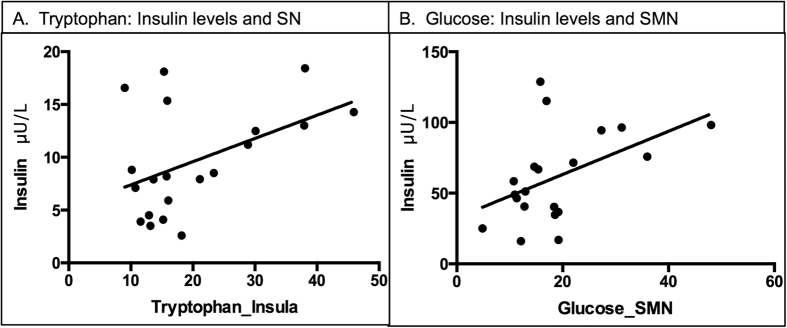
Correlations between imaging and physiological results. After L-tryptophan administration, a positive correlation (p < 0.05) was found between levels of insulin and connectivity in the saliency network (**A**). Furthermore, after glucose administration, positive correlations were present between insulin levels and connectivity in the sensorimotor network (SMN) (**B**). These results establish a link between the role of satiety hormones in amino acids synthesis and changes in functional connectivity in the brain.
